# A phase III, randomised, double‐blind, multi‐national clinical trial comparing SB12 (proposed eculizumab biosimilar) and reference eculizumab in patients with paroxysmal nocturnal haemoglobinuria

**DOI:** 10.1002/jha2.632

**Published:** 2022-12-20

**Authors:** Jun Ho Jang, Roberta Demichelis Gomez, Horia Bumbea, Larysa Nogaieva, Lily Lee Lee Wong, Soo Min Lim, Younsoo Kim, Jihye Park

**Affiliations:** ^1^ Samsung Medical Center Sungkyunkwan University School of Medicine Seoul Republic of Korea; ^2^ Instituto Nacional de Ciencias Medicas y Nutricion Salvador Zubiran Mexico City Mexico; ^3^ Bucharest Emergency University Hospital Bucharest Romania; ^4^ Cherkasy Regional Oncology Dispensary of Cherkasy Oblast Council Cherkasy Ukraine; ^5^ Queen Elizabeth Hospital Kota Kinabalu Malaysia; ^6^ Sultanah Aminah Hospital Johor Bahru Malaysia; ^7^ Samsung Bioepis Incheon Republic of Korea

**Keywords:** access, Eculizumab biosimilar, equivalence, naïve, SB12

## Abstract

Treatment of paroxysmal nocturnal haemoglobinuria (PNH) includes the monoclonal antibody eculizumab. This randomised, double‐blind, multi‐national cross‐over Phase III study in PNH patients aimed to demonstrate the equivalence of the proposed eculizumab biosimilar SB12 and reference eculizumab (Soliris, ECU). PNH patients with lactate dehydrogenase (LDH) ≥1·5× upper limit of normal were randomised into treatment sequences SB12‐ECU or ECU‐SB12. Four weekly infusions of 600 mg eculizumab were followed by fortnightly infusions of 900 mg until week 50 (ECU/SB12 cross‐over at week 26). Primary endpoints were LDH at week 26 and the time‐adjusted area under the effect curve (AUEC) of LDH over weeks 14‒26 and 40‒52. Among 46 patients (92%) who completed the study, the least squares mean (LSM) difference in LDH at week 26 (34·48; 95% confidence interval [CI] −47·66‒116·62 U/l) and geometric LSM ratio of time‐adjusted AUEC of LDH (1·08; 90% CI 0·95‒1·23) were within pre‐defined equivalence margins. Mean numbers of transfused red blood cell units, other secondary endpoints, pharmacokinetics, and pharmacodynamics were comparable. No patients developed anti‐drug antibodies. Treatment‐emergent adverse events were reported in 72% and 68% of patients in the SB12 and ECU treatment groups, respectively. The results demonstrate equivalence of SB12 to ECU and support SB12‐use in PNH patients.

## 1 INTRODUCTION

Paroxysmal nocturnal haemoglobinuria (PNH) is a rare, acquired haematopoietic disorder characterised by intravascular haemolysis [1, 2, 3]. The underlying cause is a somatic mutation in the phosphatidylinositol glycan class‐A gene that makes cells susceptible to complement‐mediated haemolysis [4]. Untreated PNH‐related chronic haemolysis can have serious consequences such as anaemia, impaired renal function, thrombosis, and pulmonary hypertension [5]. Conventional treatments, such as blood transfusions or corticosteroids are only supportive with limited impact. Bone marrow transplantation is a curative treatment but has a high risk of morbidity and mortality and requires a matching donor [6].

The monoclonal antibody eculizumab is the first approved therapy for PNH [7]. It blocks terminal complement activity by binding C5 and hence inhibits intravascular haemolysis [8, 9, 10]. Overall, eculizumab eliminates or reduces the risk of thrombosis and blood transfusion requirements, and improves anaemia, quality of life, and overall survival [11].

SB12 (Samsung Bioepis) has been developed as biosimilar to reference eculizumab (ECU; Soliris, Alexion Pharmaceuticals) and evaluated in a multi‐national, randomised clinical trial. In a Phase I study (NCT03722329), SB12 showed equivalent pharmacokinetics (PK) and comparable pharmacodynamics (PD), safety, and immunogenicity profiles to ECU [12, 13].

The multi‐national, randomised Phase III study reported here investigated the clinical equivalence of SB12 and ECU in PNH patients.

## 2 METHODS

### 2.1 Study design

This randomised, double‐blind, multi‐national, cross‐over, Phase III study (NCT04058158) was conducted at 24 study centres in eight countries between August 2019 and October 2021. The study protocol and amendments were reviewed and approved by Independent Ethics Committees or Institutional Review Boards at each study site (Supplement 1). The study was conducted in accordance with the International Council for Harmonisation and Good Clinical Practice guidelines and the Declaration of Helsinki [14]. Participants signed written informed consent forms before enrolment.

### 2.2 Patients

Eligible patients were ≥18 years with a documented diagnosis of PNH, presence of a PNH white blood cell clone (≥10% granulocyte or monocyte clone assessed by high‐sensitivity flow cytometry), and lactate dehydrogenase (LDH) levels of ≥1·5× upper limit of normal (ULN) [15]. Further inclusion criteria comprised a history of transfusion for anaemia within 12 months prior to screening or PNH‐related symptoms at screening (e.g., fatigue, haemoglobinuria, abdominal pain, chest pain, dyspnoea, dysphagia, erectile dysfunction). All patients had to be vaccinated against *Neisseria meningitidis* within 3 years prior to first dosing. Main exclusion criteria were previous complement inhibitor treatment (including ECU), a history of meningococcal disease or haematopoietic stem cell transplantation, an absolute neutrophil count ≤0·5 × 10^3^/μl, a platelet count < 70 × 10^3^/μl, and known hypersensitivity to the study drug.

### 2.3 Randomisation

Patients were randomised 1:1 to treatment sequences SB12‐ECU or ECU‐SB12 in a blinded manner. Randomisation numbers were generated by an interactive web response system.

### 2.4 Procedures

In weeks 0‒3, patients received weekly intravenous infusions of 600 mg ECU or SB12 as randomised. In weeks 4‒50, 900 mg ECU or SB12 were given every 2 weeks (Supplement 2). At week 26, patients were switched from initial ECU or SB12 to the other study drug. Periods before and after cross‐over are referred to as period 1 and 2, respectively. The end‐of‐study assessment was performed at week 52 or 58. In case of early termination, the last assessment was done 8 weeks after the last dose. Blood samples were collected before study drug administration at pre‐specified visits and analysed in the central laboratory.

### 2.5 Outcomes

The primary efficacy endpoints were reduction of haemolysis assessed by LDH levels at week 26 and time‐adjusted area under the effect curve (AUEC) of LDH from weeks 14 to 26 and 40 to 52. Secondary efficacy endpoints included the time‐course of LDH and the number of transfused packed red blood cell (pRBC) units. Other efficacy endpoints comprised changes in disease‐related laboratory parameters from baseline (e.g., LDH, PNH clone sizes, reticulocyte count, haptoglobin, and free haemoglobin), severity scores (10‐point scales) of PNH‐related symptoms over 7 days before study drug administration, breakthrough haemolysis (BTH), and major adverse vascular events (MAVEs). PK and PD endpoints comprised trough serum concentrations (C_trough_) and terminal complement activity prior to dosing. Immunogenicity endpoints comprised incidences of anti‐drug antibodies (ADAs) and neutralising antibodies (NAbs) prior to dosing. If applicable, ADAs and NAbs were also assessed at early termination visit. Safety endpoints included the incidence of adverse events (AEs), serious AEs (SAEs), and AEs of special interest (AESIs) such as infection‐related AEs and infusion‐related reactions.

### 2.6 Statistical analysis

#### 2.6.1 Equivalence margins

Pre‐defined equivalence margins were derived from meta‐analyses of a placebo‐controlled study of ECU in PNH patients (TRIUMPH) [9] and a PNH registry [16]. The equivalence margin for the two‐sided 95% confidence interval (CI) of the mean difference of LDH levels at week 26 was −1·2×ULN to 1·2×ULN (−337·2‒337·2 U/l). The equivalence margin of the two‐sided 90% CI of the mean ratio of time‐adjusted AUEC of LDH was 0·77‒1·29.

#### 2.6.2 Sample size

The sample size to detect equivalence of LDH levels at week 26 within a margin of 268 U/l at 80% power was estimated to 25 patients per treatment sequence (overall sample size of 50 patients), considering 1·2×ULN of LDH in the TRIUMPH study (= 268 U/l), no mean difference, an overall 5% significance level, and a 5% loss of patients till week 26. The same sample size was estimated for time‐adjusted AUEC of LDH considering the equivalence margin of 0·77‒1·29, a mean ratio of 1, an intra‐coefficient of variation of 42% at the overall 10% significance level, 10% loss of patients till week 52, and a power of 80%.

#### 2.6.3 Analysis sets

The modified full analysis set (M‐FAS) comprised all randomised patients who received the study drug or had at least one efficacy assessment result. The per protocol set (pps) for analysis of LDH at week 26 (PPS‐single) and for analysis of AUEC of LDH (PPS‐AUEC) comprised all patients of the M‐FAS who were treated as planned without major protocol deviations affecting efficacy assessments and had evaluable efficacy results. Populations were analysed according to their randomised treatment sequence. The safety analysis set (SAF) comprised all patients with at least one study drug administration. The PK and PD analysis sets comprised all patients of the SAF with analysis of at least one PK or PD sample, respectively.

Patients who received unplanned administration of SB12 (due to ECU shortage) were excluded from the primary efficacy analysis based on the PPS‐single and PPS‐AUEC but was included in the secondary efficacy, PK, PD, and safety analyses.

#### 2.6.4 Efficacy and safety analyses

Primary efficacy analysis of LDH levels at week 26 used a linear model with treatment and gender as fixed effects. Log_e_‐transformed LDH values were used to estimate the difference in least squares means (LSM) with 95% CI, and back transformation (delta method) provided the difference of LSM and 95% CI in original scale in PPS‐single. Analysis of time‐adjusted AUEC of LDH used a linear mixed model with treatment, sequence, period, and gender as fixed effects. Patients were nested within the sequence as a random effect. Log_e_‐transformed values of time‐adjusted AUEC of LDH were used to estimate the difference in LSM and 90% CI, and back transformation provided the ratio of geometric means and 90% CI. For sensitivity analysis, the primary efficacy analysis was repeated for the M‐FAS. In cases of study discontinuation before week 26 or 52 or insufficient values for AUEC calculation, missing data were imputed using the multiple imputation (MI) method. The number of transfused pRBCs units was compared between treatment sequences using the Wilcoxon rank‐sum test. Mean LDH time‐courses are presented by treatment sequence. All other efficacy variables were summarised by either treatment sequences and/or treatment groups. C_trough_ and values of terminal complement activities were descriptively summarised by treatment sequence within period and visit.

AEs were coded using MedDRA (Version 21.0) [17] and graded by NCI‐CTCAE v5.0 [18]. All AEs, SAEs, treatment emergent adverse events (TEAEs), serious TEAEs, and AESIs were summarised by treatment groups, frequency, and percentage. To account for ECU shortage‐related unbalanced drug exposure, an exposure adjusted event rate (EAER) was calculated by dividing the number of events with the total exposure time in patient‐years.

All statistical analyses were conducted using SAS software Version 9.4. AUEC values of LDH were calculated using WinNonlin Version 8.0.

## 3 RESULTS

**FIGURE 1:**
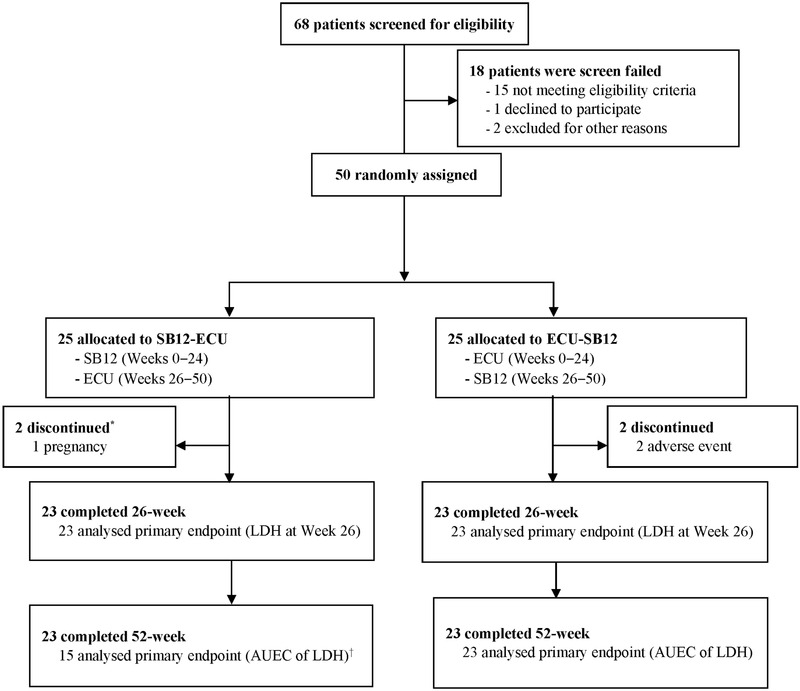
Participant Flow. ^*^One patient was withdrawn prior to administration of first study drug. ^†^Eight patients were excluded from PPS‐AUEC due to unplanned switch with SB12 due to reference eculizumab shortage. AUEC, area under the effect curve; ECU, reference eculizumab; LDH, lactate dehydrogenase; SB12, proposed eculizumab biosimilar

Among 68 screened patients, 50 were randomised to initially receive SB12 (*n* = 25) or ECU (*n* = 25) of whom 46 (92%) completed the study and four withdrew during period 1 (one before study drug administration, Figure 1). Patients who had benefited from study treatment could opt for extended, 2‐year, open‐label SB12 treatment.

During period 2, eight (16%) patients received at least one unplanned, blinded administration of SB12 instead of ECU due to a temporary ECU shortage (November 2020 to March 2021). A protocol amendment reflected these changes and a mitigation plan. All patients were informed and consented in advance to proceed with an unplanned switch of SB12.

Demographic and baseline disease characteristics results showed no statistically significant difference between the treatment sequences (Table 1). Mean age of patients was 38.1 years (range 18‒79), and most patients were male (56%). The mean (standard deviation [SD]) duration of PNH disease was 7·298 (7·5906) years among patients in the SB12‐ECU and 5·065 (6·2190) years in the ECU‐SB12 treatment sequence. Mean (SD) baseline LDH in the SB12‐ECU and the ECU‐SB12 treatment sequences were 2220·2 (2001·6) U/l and 2156·0 (1750·6) U/l, respectively.

**TABLE 1:** Baseline demographics and disease characteristics (randomised set)

	SB12‐ECU *N* = 25	ECU‐SB12 *N* = 25	Total *N* = 50
**Age (years), mean (SD)**	40·0 (13·44)	36·3 (13·67)	38·1 (13·55)
<65 years	24 (96%)	24 (96%)	48 (96%)
≥65 years	1 (4%)	1 (4%)	2 (4%)
**Gender, *n* (%)**			
Female	8 (32%)	14 (56%)	22 (44%)
Male	17 (68%)	11 (44%)	28 (56%)
**Childbearing potential** * **, *n* (%)**			
Yes	6 (75%)	12 (86%)	18 (82%)
No	2 (25%)	2 (14%)	4 (18%)
**Race, *n* (%)**			
Asian	15 (60%)	12 (48%)	27 (54%)
White	7 (28%)	11 (44%)	18 (36%)
Other	3 (12%)	2 (8%)	5 (10%)
**Ethnicity, *n* (%)**			
Hispanic or Latino	3 (12%)	2 (8%)	5 (10%)
Indian (Indian subcontinent)	2 (8%)	4 (16%)	6 (12%)
Chinese	5 (20%)	3 (12%)	8 (16%)
Other	15 (60%)	16 (64%)	31 (62%)
**Weight (kg), mean (SD)**	68·44 (14.898)	64·65 (15·777)	66·54 (15·306)
**Duration of PNH (years), mean (SD)**	7·298 (7·5906)	5·065 (6·2190)	6·182 (6·9597)
**Received a pRBC transfusion prior to screening^†^, *n* (%)**			
Yes	16 (64%)	14 (56%)	30 (60%)
No	9 (36%)	11 (44%)	20 (40%)
**Number of pRBCs units received prior to Screening^†^, mean (SD)**	6·4 (7·80)	3·8 (4·85)	5.1 (6·56)
**LDH (U/l), mean (SD)**	2220·2 (2001·6)	2156·0 (1750·6)	2188·1 (1871·9)
**Total PNH clone size of RBC (%), mean (SD)**	48·310 (26·1735)	49·686 (25·4573)	48·998 (25·5625)
**PNH clone size of granulocytes (%), mean (SD)**	89·820 (18·8196)	88·659 (12·8697)	89·240 (15·9669)
**PNH clone size of monocytes (%), mean (SD)**	92·693 (7·5677)	89·524 (12·2771)	91·109 (10·2195)
**Reticulocyte count (%), mean (SD)**	8·494 (3·7704)	7·893 (5·2386)	8·193 (4·5273)
**Haptoglobin (g/l), mean (SD)**	0·050 (0·0000)	0·050 (0·0000)	0·050 (0·0000)
**Haemoglobin (g/l), mean (SD)**	89·1 (25·04)	85·9 (19·96)	87·5 (22·47)

Abbreviations: ECU, reference eculizumab; LDH, lactate dehydrogenase; N, total number of patients in the randomised set (RAN); PNH, paroxysmal nocturnal haemoglobinuria; RBC, red blood cell; SB12, eculizumab proposed biosimilar; SD, standard deviation.

* Percentages based on the number of female patients. ^†^For 12 months prior to screening.

LDH levels at week 26 were equivalent between the SB12 and ECU treatment groups (Table 2). LSM of LDH at week 26 was 284·20 U/l and 249·72 U/l, respectively, and the estimated LSM difference (34·48; 95% CI: ‐47·66‒116·62 U/l) was within the pre‐defined equivalence margin. Sensitivity analyses results with complete cases as well as MI method were consistent with the primary analysis (34·48; 95% CI: ‐47·66‒116·62 U/l and 26·91; 95% CI: ‐56·24‒110·05 U/l, respectively) (Supplement 3). Geometric LSM of time‐adjusted AUEC of LDH in the SB12 and ECU treatment groups was 279·65 and 258·73 U/l, respectively, and the estimated ratio of geometric LSM (1·08; 90% CI: 0·95‒1·23) was within the pre‐defined equivalence margin (Table 2). Sensitivity analyses provided results consistent with the primary analysis (1·10; 90% CI: 0·99‒1·21 U/l and 1·07; 90% CI: 0·93‒1·22 U/l, respectively) (Supplement 4).

**TABLE 2:** LDH level at week 26 (PPS‐single) and time‐adjusted AUEC of LDH from weeks 14 to 26 and weeks 40 to 52 (PPS‐AUEC)

Parameter	Treatment	*n*	Least squares mean*	Difference (SB12−ECU)	
				Estimate	95% CI
LDH (U/l) at week 26	SB12	23	284·20	34·48	(−47·66*‒*116·62)
	ECU	23	249·72		

Parameter	Treatment	n	Geometric least squares mean^†^	Ratio (SB12/ECU)	
				Estimate	90% CI
Time‐adjusted AUEC of LDH (U/l)	SB12	38	279·65	1·08	(0·95*‒*1·23)
	ECU	38	258·73		

Abbreviations: CI, confidence interval; ECU, reference eculizumab; n, number of patients in the analysis set; SB12, eculizumab proposed biosimilar.

* Estimated from a linear model with log_e_‐transformed LDH at week 26 as dependent variable, and treatment and gender as fixed effects. ^†^Estimated from the linear mixed model with log_e_‐transformed time‐adjusted AUEC of LDH as dependent variable, treatment, sequence, period and gender as fixed effects and patient nested within sequence as a random effect.

**FIGURE 2:**
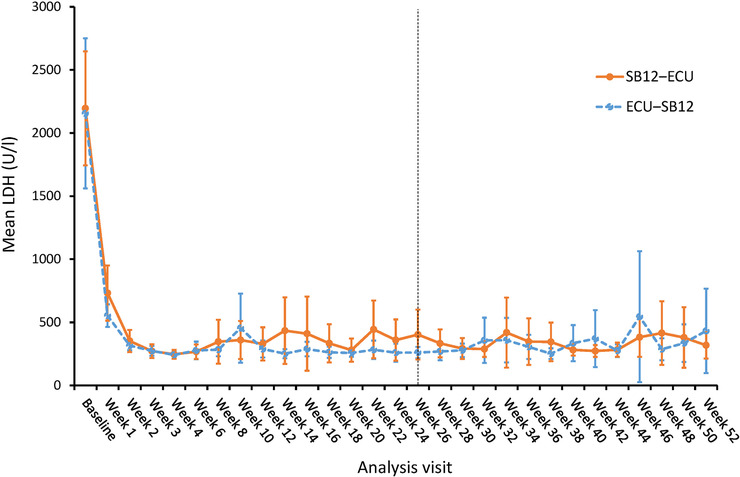
LDH profile over time (modified full analysis set). Mean LDH values are depicted as plots and whiskers represent 95% CI. The numbers of contributing patients ranged from 22 to 24 and 23 to 25 in the SB12‐ECU and ECU‐SB12 treatment sequences, respectively. CI, confidence interval; LDH, lactate dehydrogenase; ECU, reference eculizumab; SB12, proposed eculizumab biosimilar

LDH levels decreased significantly after the first dose, and mean LDH stayed below 2×ULN of LDH from week 3 onwards (Figure 2). LDH profiles were comparable between the treatment sequences throughout the study. Also, the number of transfused pRBCs units decreased in both treatment sequences after the first dose, and there was no statistically significant difference across treatment within periods (Table 3). Decrease in mean LDH, changes in other disease‐related laboratory parameters, and reductions of PNH‐related symptom severity scores from baseline to weeks 26 and 52 were comparable between the treatment sequences (Table 4).

**TABLE 3:** Transfusion records (modified full analysis set)

	SB12‐ECU *N* = 24	ECU‐SB12 *N* = 25	Total *N* = 49
**Study overall**			
*n*	24	25	49
Mean (SD)	2·9 (8·05)	2·7 (4·85)	2·8 (6·55)
Median (min‒max)	0·0 (0‒39)	0·0 (0‒22)	0·0 (0‒39)
*p‐*Value*	0·47		
**Pre‐treatment**			
n	24	25	49
Mean (SD)	0·7 (1·08)	0·8 (1·37)	0·8 (1·23)
Median (min‒max)	0·0 (0‒4)	0·0 (0‒5)	0·0 (0‒5)
*p‐*Value*	>0·9999		
**Study period 1 (prior cross‐over)**			
*n*	24	25	49
Mean (SD)	1·1 (3·72)	0·9 (2·06)	1·0 (2·96)
Median (min‒max)	0·0 (0‒18)	0·0 (0‒8)	0·0 (0‒18)
*p‐*value*	0·46		
**Study period 2 (after cross‐over)**			
*n*	23	23	46
Mean (SD)	1·1 (4·05)	1·0 (2·61)	1·1 (3·37)
Median (min‒max)	0·0 (0‒19)	0·0 (0‒12)	0·0 (0‒19)
*p‐*Value*	0·43		

*Note*: ‘Pre‐treatment’ period refers to the period from date of informed consent to first study drug administration.

* *p*‐values based on Wilcoxon rank‐sum test for difference between treatment sequences SB12‐ECU and ECU‐SB12.

Abbreviations: ECU, reference eculizumab; max, maximum; min, minimum; *N*, total number of patients in the modified full analysis set (M‐FAS); *n*, number of patients with available data in that treatment sequence; SB12, eculizumab proposed biosimilar.

**TABLE 4:** Disease‐related laboratory parameters and PNH‐related symptom severity scores (modified full analysis set)

	SB12‐ECU *N* = 24			ECU‐SB12 *N* = 25		
Timepoint	Baseline	Week 26	Week 52	Baseline	Week 26	Week 52
**Disease‐related laboratory parameter**						
LDH (U/l)	2195·2 (1069·69)	402·6 (456·73)	317·7 (241·43)	2156·0 (1141·51)	260·2 (103·67)	431·9 (773·14)
PNH clone size Type II RBC (%)	16·934 (25·8804)	19·211 (27·5523)	17·335 (25·4164)	7·594 (11·8608)	11·242 (17·3129)	11·257 (16·0857)
PNH clone size Type III RBC (%)	33·135 (20·6583)	41·412 (24·3698)	43·352 (24·9244)	42·396 (23·3097)	48·875 (29·1507)	48·812 (27·2211)
Total PNH clone size RBC (%)	49·390 (26·1616)	59·787 (23·1164)	59·933 (23·7627)	49·686 (25·4573)	61·109 (29·5036)	61·087 (28·0349)
PNH clone size Granulocytes (%)	89·628 (19·1992)	NA	87·635 (19·9793)	88·659 (12·8697)	NA	86·463 (14·9842)
PNH clone size Monocytes (%)	92·483 (7·6552)	NA	92·841 (7·9632)	89·524 (12·2771)	NA	87·152 (13·5323)
Reticulocytes (%)	8·625 (3·7925)	8·620 (4·2000)	8·458 (4·4370)	7·893 (5·2386)	7·187 (4·7444)	7·152 (3·6737)
Haptoglobin (g/l)	0·050 (0·0000)	0·110 (0·2203)	0·093 (0·1252)	0·050 (0·0000)	0·144 (0·2278)	0·093 (0·1280)
Free haemoglobin (g/l)	0·85 (0·497)	0·57 (0·435)	0·63 (0·643)	0·92 (1·166)	0·36 (0·204)	0·44 (0·298)
PNH‐related symptom severity scores^*^						
Abdominal pain	1·2 (1·95)	0·2 (0·39)	0·3 (0·69)	1·3 (2·05)	0·6 (2·10)	0·5 (1·12)
Chest pain	1·3 (1·94)	0·5 (1·7)	0·4 (1·12)	1·0 (1·81)	0·4 (0·94)	0·3 (0·65)
Dysphagia	1·3 (2·26)	0·0 (0·21)	0·1 (0·46)	0·4 (0·92)	0·0 (0·21)	0·1 (0·34)
Dyspnoea	1·8 (2·23)	0·7 (1·66)	0·5 (1·16)	1·4 (1·96)	0·9 (1·65)	0·4 (0·79)
Erectile dysfunction	1·8 (2·91)	1·1 (2·21)	0·9 (1·78)	1·6 (2·50)	0·1 (0·32)	0·1 (0·32)
Fatigue	4·2 (2·38)	1·9 (2·20)	1·7 (2·04)	3·3 (2·44)	1·9 (1·87)	2·0 (2·14)
Haemoglobinuria	3·4 (2·99)	1·1 (1·53)	0·6 (0·94)	3·1 (2·56)	0·8 (1·09)	1·0 (2·18)

*Note*: Data are presented as mean (SD). ^*^Patient‐reported severity scores (10‐point scales from 0 to 10) reflect experience for 7 days before study drug administration. A score of zero indicates that there were no symptoms at all, and 10 the worst imaginable severity.

Abbreviations: ECU, reference eculizumab; LDH, lactate dehydrogenase; *N*, total number of patients in the modified full analysis set (M‐FAS); NA, not applicable; PNH, paroxysmal nocturnal haemoglobinuria; RBC, red blood cell; SB12, eculizumab proposed biosimilar.

Overall, 25 BTH‐associated TEAEs were reported in eight (16%) patients. One MAVE (serious, grade 5 TEAE of portal vein thrombosis) was reported in the ECU treatment group (Table 5).

**TABLE 5:** Incidence of breakthrough haemolysis and major adverse vascular events by preferred term (safety set)

	SB12 *N* = 47	ECU *N* = 47	Total *N* = 49
**Breakthrough haemolysis (BTH), *n (%) E***			
Any TEAE related to BTH	8 (17%) 22	1 (2%) 3	8 (16%) 25
Haemolysis	1 (2%) 1	0 (0%) 0	1 (2%) 1
Abdominal pain	2 (4%) 4	1 (2%) 1	3 (6%) 5
Dysphagia	0 (0%) 0	1 (2%) 1	1 (2%) 1
Jaundice	2 (4%) 2	0 (0%) 0	2 (4%) 2
Haemoglobinuria	8 (17%) 15	1 (2%) 1	8 (16%) 16
**Major adverse vascular events (MAVE), *n (%) E***			
Any MAVEs	0 (0%) 0	1 (2%) 1	1 (2%) 1
Portal vein thrombosis	0 (0%) 0	1 (2%) 1	1 (2%) 1

*Note*: Percentages are based on *N* in each column.

Abbreviations: E, frequency of event; ECU, reference eculizumab; *n*, number of patients with event; *N*, total number of patients who had been treated with the respective study drug during the study; SB12, eculizumab proposed biosimilar; TEAE, treatment‐emergent adverse event.

**FIGURE 3:**
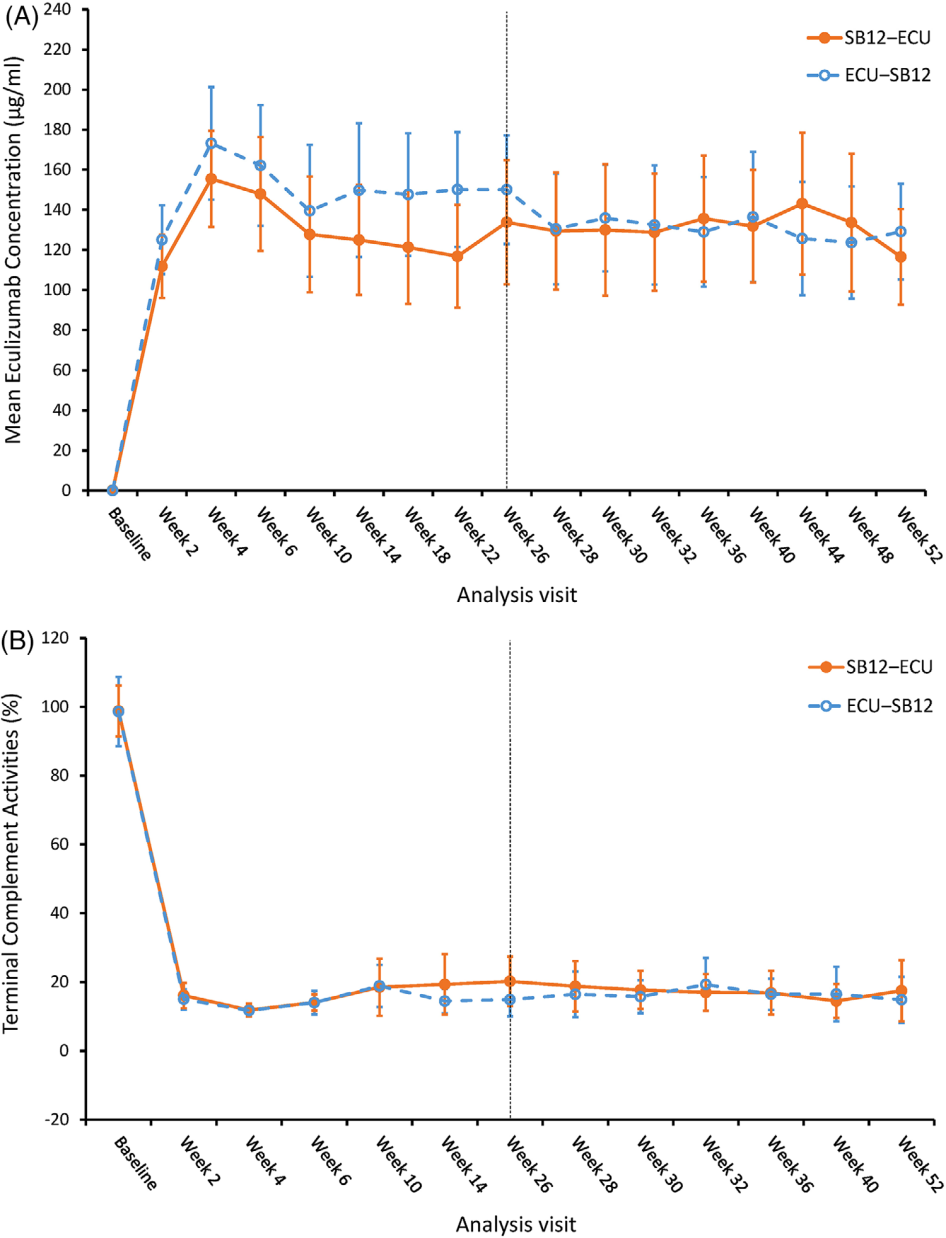
PK and PD profiles over time (pharmacokinetic analysis set and pharmacodynamic analysis set). (A) Arithmetic mean (95% CI) serum eculizumab concentrations versus nominal time plot by treatment sequences of SB12‐ECU (*n* = 24) and ECU‐SB12 (*n* = 25); whiskers represent 95% CI. The numbers of contributing patients ranged from 22 to 24 and 21 to 25 in the SB12‐ECU and ECU‐SB12 treatment sequences, respectively. (B) Arithmetic mean (95% CI) terminal complement activities (absolute value) versus nominal time plot by treatment sequences of SB12‐ECU (*n* = 24) and ECU‐SB12 (*n* = 25); whiskers represent 95% CI. The numbers of contributing patients ranged from 23 to 24 and 22 to 25 in the SB12‐ECU and ECU‐SB12 treatment sequences, respectively. CI, confidence interval; ECU, reference eculizumab; PD, pharmacodynamic; PK, pharmacokinetic; SB12, proposed eculizumab biosimilar

PK analyses showed no statistically significant difference in the time courses of mean eculizumab C_trough_ between the treatment sequences (Figure 3A). Steady‐state concentrations were achieved at week 4 for both treatments with comparable mean [95% CI] C_trough_ levels at week 26 (133·807 [102·827‒164·787] μg/ml for SB12‐ECU and 150·041 [122·961‒177·121] μg/ml for ECU‐SB12) and at week 52 (116·563 [92·707‒140·420] μg/ml for SB12‐ECU and 129·084 [105·164‒153·004] μg/ml for ECU‐SB12). PD analysis also showed no statistically significant difference in mean (95% CI) terminal complement activities at week 26 (20·24 [13·05‒27·44] % for SB12‐ECU and 14·91 [10·05‒19·77] % for ECU‐SB12) and at week 52 (17·50 [8·59‒26·41] % for SB12‐ECU and 14·87 [8·21‒21·54] % for ECU‐SB12) (Figure 3B).

Overall, 42 (86%) patients reported 218 TEAEs with comparable incidence, severity, causality, and nature between the SB12 and ECU treatment groups (Table 6). Total exposure times for SB12 and ECU were 23·4 and 19·6 patient‐years, respectively. Accordingly, EAERs were comparable (5·09 and 5·05, respectively) (Supplement 5). Most TEAEs were of grade 1 (66 events in three [6%] patients) or grade 2 (140 events in 30 [61%] patients) and not related to the study drug (198 events in 31 [63%] patients). In total, there were six serious TEAEs observed in five (10%) patients. None of three serious TEAEs reported in the SB12 treatment group was considered related to the study drug. The two of three serious TEAEs in the ECU treatment group were considered related to the study drug. All serious TEAEs were resolved except one event (cellulitis) reported in the ECU treatment group, which remained at the time of death. One patient (2%) in the ECU treatment group had a fatal TEAE of portal vein thrombosis that was considered not related to the study drug (Supplement 6).

**TABLE 6:** Safety profiles (safety set)

Variables	SB12 *N* = 47 (*n* [%])	ECU N = 47 (*n* [%])
**Patients with TEAEs, *n* (%)**	34 (72%)	32 (68%)
**Any TEAEs with incidence > 5% of patients, *n* (%)**	18 (38%)	11 (23%)
Diarrhoea	4 (9%)	2 (4%)
Corona virus infection	8 (17%)	3 (6%)
Alanine aminotransferase increased	3 (6%)	2 (4%)
Headache	2 (4%)	3 (6%)
Haemoglobinuria	8 (17%)	2 (4%)
Hypertension	3 (6%)	0 (0%)
**Patients with AESI, *n* (%)**	0 (0%)	4 (9%)
Infusion site hypersensitivity	0 (0%)	1 (2%)
Cellulitis	0 (0%)	1 (2%)
Dyspnoea	0 (0%)	1 (2%)
Rash	0 (0%)	1 (2%)
Urticaria	0 (0%)	1 (2%)
**Patients with Serious TEAEs, *n* (%)**	3 (6%)	2 (4%)
**Patients with TEAEs leading to IP discontinuation, *n* (%)**	0 (0%)	1 (2%)
**Death, *n* (%)**	0 (0%)	1 (2%)

*Note*: Percentages were based on *N* in each column.

Abbreviations: AESI, adverse event of special interest; ECU, reference eculizumab; IP, investigational product; n, number of patients with event; N, number of pooled patients who have been treated with the respective study drug during the study; SB12, eculizumab proposed biosimilar; TEAE, treatment‐emergent adverse event.

Overall, no AESI was reported in the SB12 treatment group. Five AESIs (infusion site hypersensitivity, cellulitis, dyspnoea, rash, and urticaria) in four (9%) patients were reported in the ECU treatment group. One of these patients with infusion site hypersensitivity (grade 3, considered related to the study drug) was discontinued from the study. A total of 11 COVID‐19‐related TEAEs were reported in 10 (20%) patients (SB12: eight [17%] patients; ECU: three [6%] patients) who fully recovered during the study period. EAERs of COVID‐19‐related TEAEs were 0·34 in the SB12 and 0·15 in the ECU treatment groups. All COVID‐19‐related TEAEs (all non‐serious) were considered not related to the study drug and were either grade 1 (five TEAEs in four [8%] patients) or grade 2 (six TEAEs in six [12%] patients). Three COVID‐19‐related TEAEs were reported as symptomatic (fever); the others were asymptomatic.

None of the patients had ADA‐positive results throughout the study.

## 4 DISCUSSION

Both primary endpoints, the estimated LSM difference of LDH levels at week 26, and the estimated ratio of geometric LSM of time‐adjusted AUEC of LDH from weeks 14 to 26 and 40 to 52 were within the pre‐defined equivalence margins, confirming equivalent efficacy of the proposed eculizumab biosimilar SB12 and reference eculizumab. Sensitivity analyses of the primary endpoints and analyses of secondary efficacy endpoints, PK, PD, safety, and immunogenicity consistently support equivalence of clinical efficacy. Patients in either treatment sequence (SB12‐ECU, ECU‐SB12) showed a significant reduction in mean LDH levels already 1 week after the first dose, and the levels remained within or just above the normal range during the entire study. Furthermore, consistent reductions in transfused pRBC units and severity scores of PNH‐related symptoms were achieved in both treatment sequences.

Eculizumab‐naïve patients were considered most appropriate and sensitive for the assessment of equivalence between SB12 and ECU. Eculizumab pre‐treated patients may have developed ADAs, which could influence both efficacy and safety assessments or reduce the sensitivity for detecting differences across treatment groups. Furthermore, recruitment of pre‐treated patients usually excludes patients with the lack of efficacy causing selection bias for treatment‐responsive patients.

LDH is a sensitive biochemical marker of intravascular haemolysis as LDH levels can quickly increase far above normal during periods of severe haemolysis [8]. Accordingly, LDH at a single time point was selected as primary endpoint to detect clinically meaningful differences by directly measuring intravascular haemolysis, whereas the primary endpoints of ECU's pivotal trial were stabilization of haemoglobin levels and the number of transfused pRBC units [9]. In addition, an integrated measure of LDH over time, the time‐adjusted AUEC of LDH, was defined as co‐primary endpoint to increase sensitivity for detecting potential differences between SB12 and ECU.

The cross‐over design provides additional valuable information about safety and immunogenicity after switching in a single study. Timely generation of evidence on continuous efficacy and safety after switching from ECU to a biosimilar is of high priority for patients who lose access to ECU (e.g., due to high cost or potential shortages of a medicinal product). Furthermore, in a cross‐over design, each patient serves as their own control, which should decrease variability. In fact, PK and PD profiles before and after cross‐over were comparable between treatment sequences and consistent with previous Phase I study results of SB12 [12].

The total incidence of BTH is within the range of 10·7%‒23·0% reported by trials that compared ECU with other FDA‐approved treatments [19, 20]. The higher incidence of BTH in the SB12 treatment group may be due to the ECU shortage‐related imbalanced drug exposure. Another reason may be the higher incidence of COVID‐19 and hence the delayed administration of SB12. Apart from that, all secondary and other efficacy endpoints were comparable between SB12 and ECU.

The safety profile of ECU is well documented for PNH treatment. Common AEs include headache, nasopharyngitis, nausea, pyrexia, myalgia, fatigue, and herpes simplex. There was no increase in the severity of AEs nor cumulative, irreversible toxicities reported during the study. Safety profiles of the two treatments showed no clinically meaningful differences, in accordance with the known safety profile of ECU in PNH patients, and no new safety signals.

No patient in either treatment group developed ADAs or NAbs, consistent with low ADA rates in ECU‐treated PNH populations and placebo‐controlled studies (2%‒3·4%) [9, 21, 22].

Among 50 patients, a total of eight patients had an inevitable, unplanned switch from ECU to SB12 during period 2 due to a temporary ECU‐shortage. For the primary analysis of time‐adjusted AUEC of LDH, these eight patients were excluded from the PPS‐AUEC but included in the M‐FAS. For both primary and sensitivity analyses, the 90% CI of the ratio of geometric LSM in time‐adjusted AUEC of LDH lied within the pre‐defined equivalence margin regardless of the inclusion of these patients.

Biosimilar products aim to address limited accessibility and high costs of their reference products [23]. Notably, approximately 70% of ECU‐treated PNH patients are not dosed according to the label, and two‐thirds of patients discontinue ECU within an average of 1·5 years, which may be attributed to AEs but also to the expensive treatment cost [24]. Such high discontinuation rates of a life‐saving treatment [24, 25, 26, 27] suggest a high need to improve accessibility. Indeed, the ECU‐naïve patients in this study had their PNH‐diagnosis on average 6·18 years ago, providing a real‐world example how high costs limit patients’ access to treatment. A biosimilar, such as SB12, which is comparably effective and safe as its reference product and underwent the biosimilar development pathway introduced by European Medicines Agency and the US Food and Drug Administration, can increase available treatment options and improve access to treatment and adherence. The introduction of the stringent biosimilar pathway was eminent to generate the trust and willingness of physicians to use biosimilars for patient treatment, and only products compliant with its stringent requirements such as comparative clinical equivalence studies across different countries shall be considered trusted biosimilars.

In summary, this study demonstrated equivalent clinical efficacy of the proposed eculizumab biosimilar SB12 and ECU by measuring LDH in eculizumab‐naïve PNH patients. All primary, secondary, other efficacy endpoints, PK, PD, safety, and immunogenicity profiles were comparable between SB12 and ECU, supporting the clinical use of SB12 for the treatment of PNH

## CONFLICT OF INTEREST

Roberta Demichelis Gomez has received speaker honoraria from Abbvie, Amgen, and Astellas, meeting support from Abbvie, research awards from American Society of Hematology (ASH), and participated in data safety monitoring or advisory boards of Abbvie, Amgen, Astellas, Gilead, and Teva. Horia Bumbea received speaker honoraria from Janssen, Roche, Abbvie, Novartis, Novonordisk, Sandoz, Alvogen, Amgen, Accord, Genesis, Angellini, Takeda, Bristol‐Meyers‐Squibb, Sanofi, and AstraZeneca, meeting support from Janssen, Roche, Abbvie, Novartis, Sandoz, Alvogen, Amgen, Accord, Genesis, Angellini, Takeda, Bristol‐Meyers‐Squibb, and Sobi, and participated in data safety monitoring or advisory boards of Janssen, Roche, Abbvie, Novartis, Novonordisk, Sandoz, Alvogen, Amgen, Accord, Genesis, Angellini, Takeda, Bristol‐Meyers‐Squibb, and AstraZeneca. Younsoo Kim and Jihye Park are employees of Samsung Bioepis. Jun Ho Jang, Larysa Nogaieva, Lily Lee Lee Wong, and Soo Min Lim declared no competing interests.

## ETHICS STATEMENT

The study protocol and amendments were reviewed and approved by Independent Ethics Committees or Institutional Review Boards at each study site. Participants signed written informed consent forms before enrolment. The study was registered at ClinicalTrials.gov (trial number: NCT04058158).

## Supporting information

Supplement 1: List of Investigators and Independent Ethics Committees (IECs) or Institutional Review Board (IRBs)
Supplement 2: Study design
Supplement 3: Sensitivity Analysis of Primary Efficacy Endpoint: LDH (U/l) at week 26 (modified full analysis set)
Supplement 4: Sensitivity Analysis of Primary Efficacy Endpoint: Time‐adjusted AUEC (U/l) of LDH Values (modified full analysis set)
Supplement 5: Detailed TEAE Profiles (safety set)
Supplement 6: AESI, Serious TEAEs, TEAEs Leading to IP Discontinuation or Death by Preferred Term (safety set)

## Data Availability

Upon request, and subject to certain criteria, conditions, and exceptions, Samsung Bioepis will provide access to individual de‐identified participant data to researchers whose proposals meet the research criteria and other conditions and for which an exception does not apply. Proposals should be directed to the corresponding author. For access, data requestors must enter into a data access agreement with Samsung Bioepis.
