# Supplementary material for: A phase III, randomised, double‐blind, multi‐national clinical trial comparing SB12 (proposed eculizumab biosimilar) and reference eculizumab in patients with paroxysmal nocturnal haemoglobinuria
Source: EJHaem. 2022 Dec 20;4(1):26–36. doi: 10.1002/jha2.632 (PMC9928655; doi:10.1002/jha2.632)
Supplement: Supplementary file 1 — Supplement 1: List of Investigators and Independent Ethics Committees (IECs) or Institutional Review Board (IRBs) Supplement 2: Study design Supplement 3: Sensitivity Analysis of Primary Efficacy Endpoint: LDH (U/l) at week 26 (modified full analysis set) Supplement 4: Sensitivity Analysis of Primary Efficacy Endpoint: Time‐adjusted AUEC (U/l) of LDH Values (modified full analysis set) Supplement 5: Detailed TEAE Profiles (safety set) Supplement 6: AESI, Serious TEAEs, TEAEs Leading to IP Discontinuation or Death by Preferred Term (safety set) [file JHA2-4-26-s001.docx]

**Supplement – Table of contents**

Supplement 1: List of Investigators and Independent Ethics Committees (IECs) or Institutional Review Board (IRBs) 2

Supplement 2: Study design 4

Supplement 3: Sensitivity Analysis of Primary Efficacy Endpoint: LDH (U/l) at Week 26 (modified full analysis set) 5

Supplement 4: Sensitivity Analysis of Primary Efficacy Endpoint: Time-adjusted AUEC (U/l) of LDH Values (modified full analysis set) 6

Supplement 5: Detailed TEAE Profiles (safety set) 7

Supplement 6: AESI, Serious TEAEs, TEAEs Leading to IP Discontinuation or Death by Preferred Term (safety set) 8

**Supplement 1. List of Investigators and Independent Ethics Committees (IECs) or Institutional Review Board (IRBs)**

| **IRB/IEC Name and Address** |
| --- |
| Institutional Review Board of Samsung Medical Center Samsung Medical Center  81, Irwon-ro, Gangnam-gu Seoul 06351 Korea |
| Institutional Ethics Committee for Human Research Amrita Institute of Medical Sciences and Research Centre (AIMS), AIMS Ponekkara Kochi, Kerala 682041 India |
| Fortis Memorial Research Institute Setor-44, Opposite Huda City Metro Station Gurgaon, Haryana 122002 India |
| Institutional Ethics Committee Room No 6006, 6^th^ Floor P. N Chuttani Block, PGIMER Chandigarh 160012 India |
| Institutional Ethics Committee Office of Principal, Nil Ratan Sircar (N.R.S.) Medical College and Hospital 138, A.J.C. Bose Road Centenary building Kolkata, West Bengal 700014 India |
| Ethics Committee/Research Ethics Committee Instituto Nacional de Ciencias Médicas y Nutrició Salvador Zubirán  Av. Vasco de Quiroga No 15,  Col Belisario Dominguez sección XVI Tialpan Mexico City 14080 Mexico |
| National Bioethics Committee for Medicine and Medical Devices 19-21 Stefan cel Mare Road Bucharest 020125 Romania |
| The Ethics Commission at Communal nonprofit enterprise “Vinnytsya regional Clinical Hospital named after N. I. Pirogov ofVinnytsia Regional Council” 46, Pyrohova Street Vinnytsia 21018 Ukraine |
| The Ethics Commission at Communal Nonprofit Enterprise Cherkasy Regional Oncology Dispensary of Cherkasy Oblast Council 7 Mendeleieva Street Cherkasy 18009 Ukraine |
| The Ethics Commission at Public Enterprise Poltava Regional Clinical Hospital Named After M.V. Sklifosovskyi of Poltava Regional Council  23, Shevchenka Street Poltava 36011 Ukraine |
| The Ethics Commission at Communal Non-profit Enterprise Regional Center of Oncology 4 Lisoparkivska Street Kharkiv 61070 Ukraine |
| Medical Research and Ethics Committee (MREC) Secretariat of National Institutes of Health (NIHSEC) c/o Blok A, Kompleks Institut Kesihatan Negara (NIH)  No. 1, Jalan Setia Murni 13/52 Seksyen U13, Bandar Setia Alam Shah Alam, Selangor 40170 Malaysia |
| Chang Gung Medical Foundation Institutional Review Board 199, Tung Hwa North Road Taipei 10507 Taiwan  Chang Gung Medical Foundation – Linkou Branch (Previous) Hematology Examination Room 6F, Pathology Building, No.5, Fu-Hsing Street, Kuei Shan Dist. Taoyuan City, 333 Taiwan |
| Tri-service General Hospital Independent review board No. 325, Sector 2, Cheng-Kung Road., NeiHu Taipei 114 Taiwan |
| National Cheng Kung University Hospital Independent review board No. 138 Sheng-Li Road Tainan 70403 Taiwan |
| Central Research Ethics Committee (CREC) 3rd Floor, NRCT 3 Building, Office of the National Research Council of Thailand (NRCT), 196, Subdivision 5, Phahonyothin, Lat Yao Chatuchak, Bangkok 10900 Thailand |
| The Khon Kaen University Ethics Committee in Human Research Room 5317, 3rd Floor Wadwichakarn Building Faculty of Medicine, Khon Kaen University Muang, Khon Kaen 40002 Thailand |
| The Institutional Review Board of the Faculty of Medicine, Chulalongkorn University 3rd Floor, Anuntamahidol Building, Rama IV Road Pathumwan, Bangkok 10330 Thailand |

Supplement 2: Study design. In Weeks 0‒3 (W0‒W3), patients received weekly intravenous infusions of 600 mg ECU or SB12 as randomised. In Weeks 4-50 (W4‒W50), 900 mg ECU or SB12 were given every two weeks. At Week 26 (W26), patients were switched from initial ECU or SB12 to the other study drug. Periods before and after cross-over are referred to as Period 1 and 2, respectively.

AUEC, area under the effect curve; ECU, reference eculizumab; PNH, paroxysmal nocturnal haemoglobinuria; R, randomisation; SB12 proposed eculizumab biosimilar; W, week.


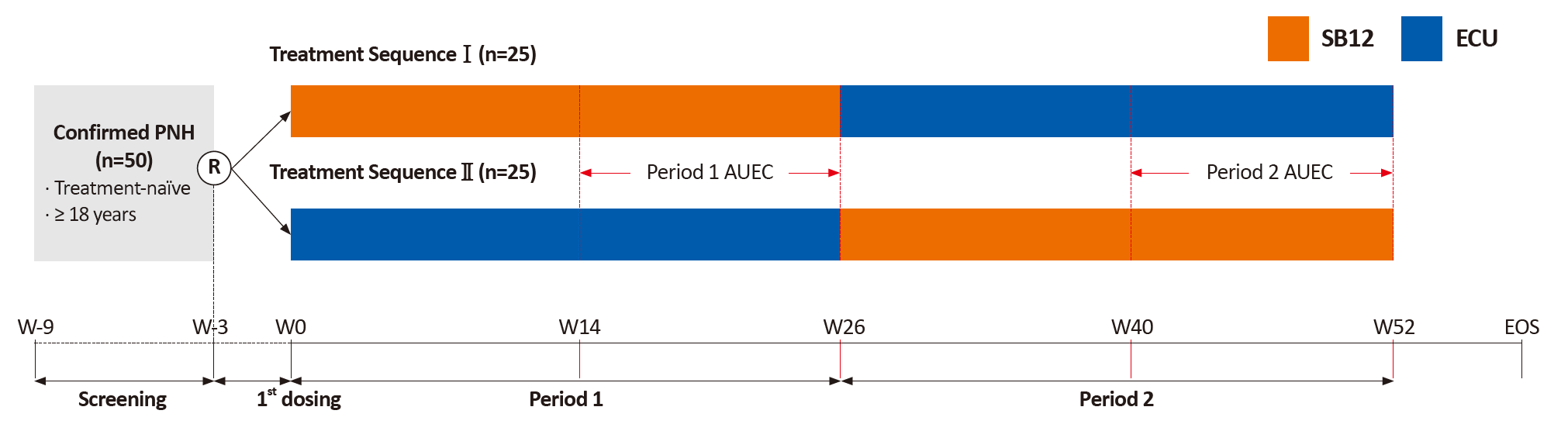


**Supplement 3. Sensitivity Analysis of Primary Efficacy Endpoint: LDH (U/l) at Week 26 (modified full analysis set)**

| **Parameter** | **Treatment** | **n** | **Least Squares Mean^*^** | **Difference (SB12-ECU)** | |
| --- | --- | --- | --- | --- | --- |
|  |  |  |  | **Estimate** | **95% CI** |
| **Method: Complete case analysis** | | | | | |
| LDH (U/l) at Week 26 | SB12 (N=24) | 23 | 284·20 | 34·48 | (−47·66‒116·62) |
|  | ECU (N=25) | 23 | 249·72 |  |  |
| **Method: Multiple imputation** | | | | | |
| LDH (U/l) at Week 26 | SB12 (N=24) | 24 | 282·87 | 26·91 | (−56·24‒110·05) |
|  | ECU (N=25) | 25 | 255·96 |  |  |

^*^ Least squares mean (LSM) difference and its 95% CI in original scale were obtained by applying delta method to the LSM ratio estimated from a linear model with natural log-transformed LDH at Week 26 as dependent variable, and treatment and gender as fixed effects.

CI, confidence interval; ECU, reference eculizumab; LDH, lactate dehydrogenase; n, number of patients in the analysis; N, total number of patients who had been treated with SB12 or ECU in Period 1, respectively; SB12, eculizumab proposed biosimilar.

**Supplement 4. Sensitivity Analysis of Primary Efficacy Endpoint: Time-adjusted AUEC (U/l) of LDH Values (modified full analysis set)**

| **Parameter** | **Treatment** | **n** | **Geometric Least Squares Mean^*^** | **Ratio (SB12/ECU)** | |
| --- | --- | --- | --- | --- | --- |
|  |  |  |  | **Estimate** | **90% CI** |
| **Method: Complete case analysis** | | | | | |
| Time-adjusted AUEC (U/l) of LDH | SB12 (N=49) | 46 | 290·88 | 1·10 | (0·99‒1·21) |
|  | ECU (N=49) | 46 | 265·37 |  |  |
| **Method: Multiple imputation** | | | | | |
| Time-adjusted AUEC (U/l) of LDH | SB12 (N=49) | 49 | 290·09 | 1·07 | (0·93‒1·22) |
|  | ECU (N=49) | 49 | 271·62 |  |  |

^*^ Geometric means ratio and 90% CI was obtained by performing back transformation of least squares mean difference and its 90% CI from the linear mixed model with natural log-transformed time-adjusted AUEC of LDH as dependent variable, and treatment, sequence, period and gender as fixed effects and patient nested within sequence as a random effect.
AUEC, area under the effect curve; CI, confidence interval; ECU, reference eculizumab; LDH, lactate dehydrogenase; n, number of patients in the analysis; N, total number of pooled patients in the M-FAS who belonged to SB12 or ECU treatment group in either Period 1 or 2, respectively; SB12, eculizumab proposed biosimilar.

**Supplement 5. Detailed TEAE Profiles (safety set)**

| **Variables** | **SB12 (N=47)** | | **ECU (N=47)** | | **Total (N=49)** | |
| --- | --- | --- | --- | --- | --- | --- |
|  | **Person years=23·4** | | **Person years=19·6** | | **Person years=43·1** | |
|  | **n (%)** | **E [EAER]** | **n (%)** | **E [EAER]** | **n (%)** | **E [EAER]** |
| **TEAEs** | | | | | | |
| Any TEAEs | 34 (72%) | 119 [5·09] | 32 (68%) | 99 [5·05] | 42 (86%) | 218 [5·06] |
| **TEAEs by severity** | | | | | | |
| Grade 1 | 1 (2%) | 38 [1·62] | 6 (13%) | 28 [1·43] | 3 (6%) | 66 [1·53] |
| Grade 2 | 27 (57%) | 73 [3·12] | 23 (49%) | 67 [3·42] | 30 (61%) | 140 [3·25] |
| Grade 3 | 6 (13%) | 8 [0·34] | 2 (4%) | 3 [0·15] | 8 (16%) | 11 [0·26] |
| Grade 4 | 0 (0%) | 0 [0·00] | 0 (0%) | 0 [0·00] | 0 (0%) | 0 [0·00] |
| Grade 5 | 0 (0%) | 0 [0·00] | 1 (2%) | 1 [0·05] | 1 (2%) | 1 [0·02] |
| **TEAEs by causality** | | | | | | |
| Related | 3 (6%) | 5 [0·21] | 8 (17%) | 15 [0·77] | 11 (22%) | 20 [0·46] |
| Not related | 31 (66%) | 114 [4·87] | 24 (51%) | 84 [4·29] | 31 (63%) | 198 [4·59] |
| **TEAEs by outcome** | | | | | | |
| Recovered/Resolved | NA | 103 [4·4] | NA | 84 [4·29] | NA | 187 [4·34] |
| Recovered/Resolved with sequelae | NA | 0 [0·00] | NA | 2 [0·10] | NA | 2 [0·05] |
| Recovering/Resolving | NA | 3 [0·13] | NA | 5 [0·26] | NA | 8 [0.19] |
| Not recovered/Not resolved | NA | 11 [0·47] | NA | 7 [0·36] | NA | 18 [0.42] |
| Fatal | NA | 0 [0·00] | NA | 1 [0·05] | NA | 1 [0·02] |
| Unknown | NA | 2 [0·09] | NA | 0 [0·00] | NA | 2 [0·05] |
| **AESI** | | | | | | |
| Any AESI | 0 (0%) | 0 [0·00] | 4 (9%) | 5 [0·26] | 4 (8%) | 5 [0·12] |
| **Serious TEAE** | | | | | | |
| Any Serious TEAEs | 3 (6%) | 3 [0·13] | 2 (4%) | 3 [0·15] | 5 (10%) | 6 [0·14] |
| **TEAEs related to COVID-19 infection** | | | | | | |
| Any TEAEs for COVID-19 | 8 (17%) | 8 [0·34] | 3 (6%) | 3 [0·15] | 10 (20%) | 11 [0·26] |
| Any Serious TEAEs for COVID-19 | 0 (0%) | 0 [0·00] | 0 (0%) | 0 [0·00] | 0 (0%) | 0 [0·00] |

Percentages are based on N in each column. An event is reported under the treatment the patient was last received prior to the event. If a patient had multiple events with different severity (or causality), then the patient was counted only once at the worst severity (or causality) for the number of patients (n).
AESI, adverse event of special interest; COVID-19, Coronavirus Disease 2019; E, frequency of TEAE; EAER, exposure-adjusted event rate; ECU, reference eculizumab; n, number of patients with event; N number of pooled patients who have been treated with the respective study drug during the study (N for Total represents the total number of patients in the safety set); NA, not applicable; SB12, eculizumab proposed biosimilar; TEAE, treatment-emergent adverse event.

**Supplement 6. AESI, Serious TEAEs, TEAEs Leading to IP Discontinuation or Death by Preferred Term (safety set)**

| **Variables** | **SB12 (N=47)** | | **ECU (N=47)** | | **Total (N=49)** | |
| --- | --- | --- | --- | --- | --- | --- |
|  | **Person years=23·4** | | **Person years=19·6** | | **Person years=43·1** | |
|  | **n (%)** | **E** | **n (%)** | **E** | **n (%)** | **E** |
| **AESI by PT** | | | | | | |
| Infusion site hypersensitivity | 0 (0%) | 0 | 1 (2%) | 1 | 1 (2%) | 1 |
| Cellulitis | 0 (0%) | 0 | 1 (2%) | 1 | 1 (2%) | 1 |
| Dyspnoea | 0 (0%) | 0 | 1 (2%) | 1 | 1 (2%) | 1 |
| Rash | 0 (0%) | 0 | 1 (2%) | 1 | 1 (2%) | 1 |
| Urticaria | 0 (0%) | 0 | 1 (2%) | 1 | 1 (2%) | 1 |
| **Serious TEAEs by PT** | | | | | | |
| Haemolysis | 1 (2%) | 1 | 0 (0%) | 0 | 1 (2%) | 1 |
| Infusion site hypersensitivity | 0 (0%) | 0 | 1 (2%) | 1 | 1 (2%) | 1 |
| Portal vein thrombosis | 0 (0%) | 0 | 1 (2%) | 1 | 1 (2%) | 1 |
| Wound infection bacterial | 1 (2%) | 1 | 0 (0%) | 0 | 1 (2%) | 1 |
| Cellulitis | 0 (0%) | 0 | 1 (2%) | 1 | 1 (2%) | 1 |
| Hand fracture | 1 (2%) | 1 | 0 (0%) | 0 | 1 (2%) | 1 |
| **TEAEs leading to IP discontinuation by PT** | | | | | | |
| Infusion hypersensitivity | 0 (0%) | 0 | 1 (2%) | 1 | 1 (2%) | 1 |
| **TEAEs leading to death by PT** | | | | | | |
| Portal vein thrombosis | 0 (0%) | 0 | 1 (2%) | 1 | 1 (2%) | 1 |

Percentages are based on N in each column. An event is reported under the treatment the patient was last received prior to the event. If a patient had multiple events with different severity (or causality), then the patient was counted only once at the worst severity (or causality) for the number of patients (n).
AESI, adverse event of special interest; E, frequency of TEAE; ECU, reference eculizumab; IP, investigational product; n, number of patients with event; N number of pooled patients who have been treated with the respective study drug during the study (N for Total represents the total number of patients in the safety set); PT, preferred term; SB12, eculizumab proposed biosimilar; TEAE, treatment-emergent adverse event.
